# Improved fatty acid composition of field cress (*Lepidium campestre*) by CRISPR/Cas9-mediated genome editing

**DOI:** 10.3389/fpls.2023.1076704

**Published:** 2023-01-18

**Authors:** Sjur Sandgrind, Xueyuan Li, Emelie Ivarson, Eu Sheng Wang, Rui Guan, Selvaraju Kanagarajan, Li-Hua Zhu

**Affiliations:** Department of Plant Breeding, Swedish University of Agricultural Sciences, Lomma, Sweden

**Keywords:** CRISPR-editing, field cress, oil quality, protoplast, transgene-free mutants

## Abstract

The wild species field cress (*Lepidium campestre*) has the potential to become a novel cover and oilseed crop for the Nordic climate. Its seed oil is however currently unsuitable for most food, feed, and industrial applications, due to the high contents of polyunsaturated fatty acids (PUFAs) and erucic acid (C22:1). As the biosynthesis of these undesirable fatty acids is controlled by a few well-known major dominant genes, knockout of these genes using CRISPR/Cas9 would thus be more effective in improving the seed oil quality. In order to increase the level of the desirable oleic acid (C18:1), and reduce the contents of PUFAs and C22:1, we targeted three important genes *FATTY ACID ELONGASE1* (*FAE1*), *FATTY ACID DESATURASE2* (*FAD2*), and *REDUCED OLEATE DESATURASE1 (ROD1*) using a protoplast-based CRISPR/Cas9 gene knockout system. By knocking out *FAE1*, we obtained a mutated line with almost no C22:1, but an increase in C18:1 to 30% compared with 13% in the wild type. Knocking out *ROD1* resulted in an increase of C18:1 to 23%, and a moderate, but significant, reduction of PUFAs. Knockout of *FAD2*, in combination with heterozygous *FAE1fae1* genotype, resulted in mutated lines with up to 66% C18:1, very low contents of PUFAs, and a significant reduction of C22:1. Our results clearly show the potential of CRISPR/Cas9 for rapid trait improvement of field cress which would speed up its domestication process. The mutated lines produced in this study can be used for further breeding to develop field cress into a viable crop.

## 1 Introduction

Population growth is driving an increase in the demand for vegetable oils for food, feed, and industrial applications. Furthermore, there is overwhelming evidence that the use of fossil fuels is a major cause of climate change, which could be partially mitigated by exchanging fossil fuels with sustainably growing biofuels (IPCC, 2011). Developing novel oilseed crops that can be grown without adverse effects on land use could be a part of the solution. Field cress (*Lepidium campestre*) is a wild biennial species in the Brassicaceae family that has been targeted for domestication as a novel cover and oilseed crop under sown with a spring cereal. After the harvest of the cereal in the summer, field cress could prevent nutrient leaching and soil erosion until harvest next season. It has excellent cold-hardiness, a high seed yield potential, branching in only upper parts of upright stems, good seed size, and is resistant to the pollen beetle (Merker et al., 2010). These characteristics make it a promising crop for cultivation in temperate zones such as the Nordic region, where it could increase land productivity while providing beneficial ecosystem services. However, some traits need to be improved before the species becomes an economically viable crop.

The high levels of linoleic acid (C18:2), linolenic acid (C18:3), and erucic acid (C22:1) render the oil unsuitable for food and some industrial uses. Polyunsaturated fatty acids (PUFAs) are oxidatively unstable and have low melting points with reduced shelf life and suitability for uses such as fuel and frying (Singh et al., 2019). C22:1 is an important fatty acid for the chemical industry, but is considered potentially toxic in food and feed at high levels. According to the European Union Commission Regulation 2019/1870 (EU, 2019), the erucic acid content in foodstuffs may not exceed 2%. Oleic acid (C18:1) has higher oxidative stability, being suitable for some industrial applications on one hand, and its food consumption is also linked to health benefits (Sales-Campos et al., 2013) on the other hand. Improving the oil quality by increasing C18:1, and reducing the content of PUFAs and C22:1 is thus an important goal in developing field cress into a viable food crop.

In plants, the primary biosynthesis of fatty acids occurs in plastids, with the resulting fatty acids being exported into the cytosol predominantly as C18:1, along with smaller amounts of palmitic acid (C16:0) and stearic acid (C18:0) (Bates et al., 2013). Further modification of C18:1 occurs on the endoplasmic reticulum, mainly *via* elongation to eicosenoic acid (C20:1) and C22:1 by fatty acid elongase 1 (FAE1), or desaturation to C18:2 by fatty acid desaturase 2 (FAD2), which can be further desaturated to C18:3 by fatty acid desaturase 3 (FAD3) (McCartney et al., 2004; Haslam and Kunst, 2013). Knockdown or knockout of *FAE1* has been shown to dramatically reduce the production of C20:1 and C22:1 in multiple species, causing an increase of C18:1, C18:2, and C18:3. Similarly, knockdown or knockout of *FAD2* has been shown to reduce the content of PUFAs and increase the content of C18:1 (Li et al., 2016; Ozseyhan et al., 2018; Do et al., 2019; Chopra et al., 2020; Huang et al., 2020; Jarvis et al., 2021; Liu et al., 2022). In field cress, RNAi knockdown of *FAE1* and *FAD2* resulted in lines with dramatically improved oil quality, up to 80% C18:1, compared with 11% in the wild type. Furthermore, the C18:3 content was reduced from 40% to 3%, and C22:1 was decreased from 20% to 0.1% (Ivarson et al., 2016).

One major concern with the knockdown or knockout of the *FAD2* gene has been its potential adverse physiological effects on growth and development. In this regard, the *REDUCED OLEATE DESATURASE1 (ROD1)* gene might be a good alternative, which has been shown to be involved in regulating the C18:1 content, while no adverse effects on growth, flowering time and seed number of pennycress (Jarvis et al., 2021). The gene encodes phosphatidylcholine:diacylglycerol phosphotransferase (PDCT), which directs C18:1 to further desaturation by catalyzing interconversion between phosphatidylcholine and diacylglycerol (Bates, 2016). Knockdown or knockout of *ROD1* has been shown to significantly reduce the PUFAs content, and increase the C18:1 content in several oilseed species (Lu et al., 2009; Guan et al., 2015; Bai et al., 2020; Jarvis et al., 2021).

CRISPR/Cas9-mediated gene editing holds great promise for rapid improvement of the seed oil quality of oil crops, as disruption of even a single gene can have a significant impact on the fatty acid profile of seed oil and provides new opportunities to enhance oil production in vegetative tissues. Applications of the CRISPR technique in modifying fatty acid profiles and increase the oil production in vegetative tissues have been reviewed by Park and Kim (2022). For instance, knocking out of *FAD2* could increase the oleic acid contents in some species, while knocking out of some paralogs of the *FATB* gene reduced the levels of saturated fatty acids in soybean and peanuts. With help of the CRISPR technique, it is also possible to regulate negative regulatory elements in the upstream sequences of a target gene for enhancing expression of the target gene. For instance, Bhunia et al. (2022) has reported that enhanced expression of *DGAT2* under the control of a promoter from an upstream gene deleting the intervening genomic sequence using dual-guide CRISPR/Cas9 was obtained, which resulted in increased TAG content in the leaves of Arabidopsis (Bhunia et al., 2022).

Field cress is diploid (2*n* = 2*x* = 16) with a relatively small genome size (Rice et al., 2015), and it is expected that many genes share high sequence similarity with the model species *Arabidopsis thaliana* as we have shown from our previous studies (Ivarson et al., 2016). As such, cloning and sequencing of target genes are relatively straightforward. Furthermore, our group has very recently developed and published efficient protoplast protocols for oilseed crops including field cress (Li et al., 2021; Sandgrind et al., 2021), thus enabling the generation of transgene-free CRISPR-edited mutants.

In this study, we aimed at demonstrating that CRISPR/Cas9 can be an efficient genome editing tool for trait improvement of field cress, and for developing transgene-free mutants with improved oil quality by knocking out key fatty acid biosynthesis genes.

## 2 Materials and methods

### 2.1 Plant material and growth conditions

The seeds of field cress (*L. campestre* L.) used in this study were originated from accession no. 94-7, collected in Öland, Sweden, and multiplied in greenhouse or biotron.

Seeds were surface sterilized with 15% (w/v) calcium hypochlorite (Ca(ClO)_2_) and Tween 20 for 20 min, and subsequently rinsed with sterile water. Sterilized seeds were planted on the germination medium (half-strength MS, 10 g/L sucrose, 7 g/L Bacto Agar, pH 5.7) in sterile and single-use plastic boxes.

*In vitro* cultures were maintained in a climate chamber with a temperature of 23°C/18°C (day/night) and 16 h photoperiod with a light intensity of 40 µmol m^-2^ s^-1^.

To induce flowering, the plantlets were vernalized *in vitro* at 4°C for eight weeks. Thereafter, they were planted in soil and grown in biotron with a temperature of 21°C/16°C (day/night), 16 h photoperiod, light intensity of 250 µmol m^-2^ s^-1^, and 60% humidity. Mature seeds were harvested and stored at 4°C.

### 2.2 Cloning of *LcROD1*

The *AtROD1* gDNA sequence (NM_112452) was used for a BLAST query in the WGS field cress database in NCBI to find an *A. thaliana* ortholog in field cress (WJSH01021407). Using the sequence of this ortholog, we designed primers (**Supplementary Table S1**) to amplify and sequence the full length *LcROD1* according to Muthusamy et al., 2020.

### 2.3 Design of sgRNAs and preparation of CRISPR/Cas9 vectors

**Figure 1:**
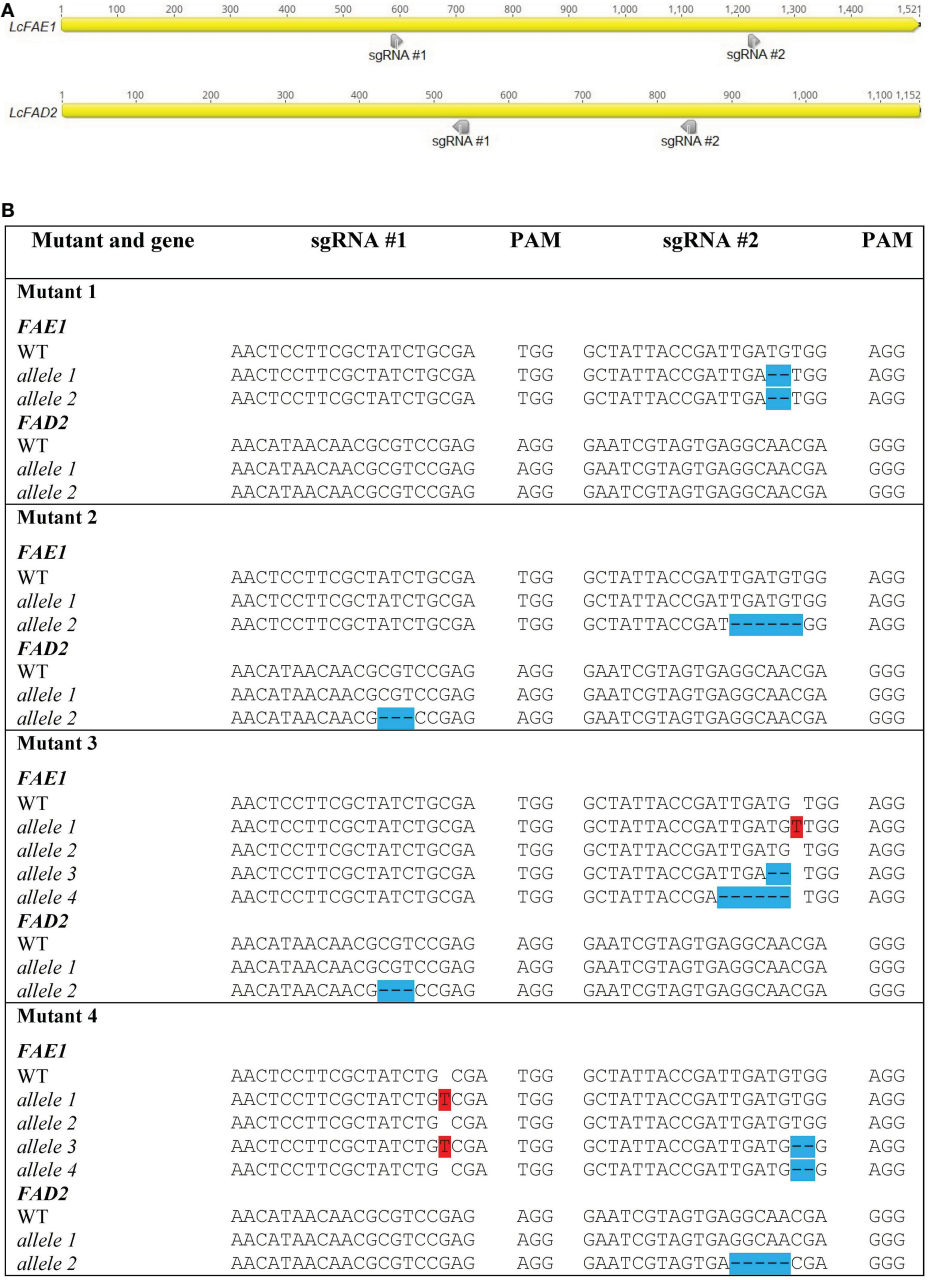
Locations of sgRNAs selected **(A)** and types of mutations detected in *FAE1* and *FAD2* in different mutant lines **(B)**. Insertions are highlighted in red. Deletions are highlighted in blue. (See also chromatographs from Sanger sequencing in **Supplementary Figure S4**).

*LcFAE1* and *LcFAD2* sequences were obtained from the NCBI GenBank database (FJ907545 and FJ907546, respectively), and the sequences were verified in the genotype used by cloning and sequencing. Based on location in the target genes, GC content, absence of TTTT motifs, and starting nucleotide, two sgRNAs targeting *FAE1*, two sgRNAs targeting *FAD2*, and four sgRNAs targeting *ROD1* were selected using the CRISPOR (Concordet and Haeussler, 2018) and CRISPRdirect (Naito et al., 2015) programs (**Figure 1**).

The selected sgRNA sequences were integrated into individual sgRNA cloning vectors containing either AtU3d (Addgene plasmid #66201), AtU3b (Addgene plasmid #66198), AtU6-1 (Addgene plasmid #66202), or AtU6-29 (Addgene plasmid #66203) promoter (**Supplementary Table S2**) using the primers listed in **Supplementary Table S3**. Finally, the sgRNA expression cassettes were sequentially ligated into pYLCRISPR/Cas9P_ubi_-N vectors according to the protocol by Ma et al. (2015).

Two CRISPR/Cas9 vectors were constructed. One contained two sgRNAs targeting *FAE1* and two sgRNAs targeting *FAD2*, and another contained four sgRNAs targeting *ROD1*, designated pYLCRISPR/Cas9P_ubi_-N_FAE1/FAD2 and pYLCRISPR/Cas9P_ubi_-N_ROD1, respectively (**Supplementary Table S2**).

### 2.4 Protoplast isolation, transfection, and culture

Protoplast isolation, transfection, and culture were performed according to our previously published protocol (Sandgrind et al., 2021), with minor modifications. Approximately 150 000 to 200 000 protoplasts in 200 µl 0.5 M mannitol were gently mixed with 40 µg vector DNA (1 µg/µl) and 240 µl freshly prepared PEG-calcium solution (25% (w/v) PEG4000, 0.4 M mannitol, 0.1 M CaCl_2_), and incubated for 5 min, followed by centrifugation and washing. The transfected protoplasts were then cultured *in vitro* in different media for cell division, callus and shoot formation according to the protocol.

### 2.5 Identification of mutant lines

Leaf samples were taken from *in vitro* regenerated shoots and crushed in Phire Dilution Buffer (Thermo Scientific) to extract gDNA. The supernatant was used directly as template for PCR amplification with the sequence-specific primers, in which the forward primers were fluorescently labeled, using Phusion High-Fidelity PCR Master Mix with HF Buffer (Thermo Scientific) (**Supplementary Table S4**). The PCR amplicons were subsequently subjected to high-resolution fragment analysis (HRFA) as described by Andersson et al. (2017). To confirm the types of mutations identified by HRFA, PCR amplicons obtained with non-fluorescent primers were ligated into pJET1.2/blunt Cloning vector (Thermo Scientific) and transformed into *E. coli* (Stellar Competent Cells, Takara Bio). Single colonies were randomly selected and analyzed by Sanger sequencing (Eurofins Genomics or LGC Genomics).

Once the regenerated shoots were confirmed to be mutated by sequencing, they were transferred to the rooting medium according to the protocol by Ivarson et al. (2013). Rooted shoots were subsequently vernalized *in vitro* for floral induction, transplanted to soil, and grown to maturity in biotron under the conditions as stated above.

All target sites were sequenced to examine if transgene integration had occurred, and PCR was performed on the mutant lines using *Cas9-*specific primers (**Supplementary Table S5**).

### 2.6 Analysis of fatty acid composition for phenotyping

Pooled seed analysis of homozygous T_0_ mutants

Pooled seeds were used for lipid extraction and analysis using the method described by Ivarson et al. (2016), with minor modifications. Ten randomly selected mature seeds per sample were ground in 1 ml 0.15 M HAc and 3.75 ml CHCl_3_/MeOH using an Ultra Turrax immersion blender. After homogenization, 1.25 ml CHCl_3_ and 0.9 ml H_2_O were added, vortexed and then transferred to tubes for centrifugation for 2 min at 3000 rpm. Thereafter, 200 µl of the CHCl_3_ phase was transferred to a test tube and dried under an N_2_ beam. For methylation, 100 µl heptane and 2 ml methylation solution (2% H_2_SO_4_ in methanol) were added into the tubes and capped, the tubes were then methylated at 95°C for 1 h. After cooling, 1 ml H_2_O and 0.75 ml heptane were added, vortexed, and centrifuged for 2 min at 2000 rpm. Finally, 100 µl heptane phase was transferred to a GC vial with insert, from which 1 µl was used for analysis on a GC machine (Agilent 7890A) with a WCOT Fused Silica CP-Wax 58 column and flame-ionization detection (GC-FID) with the program of split ratio 10:1, 150^0^C for 0.2 min, 4^0^C/min to 210^0^C, 10^0^C/min to 250^0^C and hold at 250^0^C for 5 min. Peaks were identified according to their retention times compared with a standard FAME mixture, and the results were expressed as the area percentage of the methyl esters in total detectable peak areas. Three biological samples were analyzed per mutant line.

Half-seed analysis of heterozygous T_0_ mutants

Individual seeds from heterozygous T_0_ mutated lines and wild type were first phenotyped by analyzing the fatty acid profiles with GC-FID, as stated above. The interesting phenotypes were subsequently genotyped by Sanger sequencing. Half-seed analysis was based on the protocol by Li et al. (2012). Seeds were surface sterilized, and the larger outer cotyledons were excised for fatty acid analysis. The remaining parts of the seeds were germinated *in vitro* and the leaves were used for genotyping by sequencing. Excised cotyledons were ground with mortar and pestle in 500 µl heptane, followed by adding 500 µl heptane. The solution was then filtered through a glass Pasteur pipette with a glass wool plug into a test tube and dried under an N_2_ beam. Thereafter, 100 µl heptane and 2 ml methylation solution (2% H_2_SO_4_ in methanol) were added, and the sample methylated at 95°C for 45 min. After cooling down, 0.5 ml heptane and 2 ml water were added, and the samples were then vortexed and centrifuged for 2 min at 2000 rpm. Finally, 100 µl of the heptane phase was transferred to a GC vial with insert, and 1 µl was analyzed on the GC machine as described for pooled seed analysis. Peaks were identified, and results were expressed as described for pooled seed analysis.

### 2.7 Statistical analysis

Data for fatty acid profile analysis (*n* = 3) was analyzed with RStudio 2021.09.1 Build 372, using the nlme and emmeans packages. ANOVA analysis was carried out with Tukey’s test at 95% confidence level.

## 3 Results

### 3.1 Cloning results of *LcROD1*

The field cress ortholog WJSH01021407 showed 80% of sequence homology with the full sequence of *AtROD1* (NM_112452) from *A. thaliana*. Cloning and sequencing of the gene from gDNA and cDNA revealed that the gene consists of three exons (399, 138, and 327 nt, respectively, in total 864 nt). The *LcROD1* gene shared 87% sequence homology with the *AtROD1* coding sequence, and two introns (513 and 416 nt, respectively, in total 929 nt) (**Supplementary Figure S1**). The *LcROD1* sequence is now available at GenBank with the accession number OP703165.1.

### 3.2 Molecular screening of mutants

#### 3.2.1 Identification of *fae1* and *fad2* mutants

HRFA analysis of 268 T_0_ regenerated shoots derived from protoplasts transfected with the vector DNA (pYLCRISPR/Cas9P_ubi_-N_FAE1/FAD2) targeting *FAE1* and *FAD2* identified 11 mutated lines, indicating an estimated 4.1% mutation efficiency. The sequencing results confirmed four distinct mutant genotypes, named Mutant 1, 2, 3, and 4, respectively (**Figure 1**). All four sgRNAs were found to be able to induce mutations. One homozygous mutated *fae1* line (Mutant 1) was obtained in T_0_, while no homozygous *fad2* mutants or double *fae1fad2* mutants were detected in T_0_. Mutant 2 had heterozygous in-frame mutations in both *FAE1* and *FAD2*. Sequencing revealed that Mutant 3 and 4 contained more than two *FAE1* alleles, indicating chimerism. In Mutant 3, a wild type and three mutated *FAE1* alleles were detected, of which two mutated alleles were predicted to have disruptions to the open reading frame, while one mutation was in-frame. In *FAD2*, two distinct alleles were detected, a wild type allele and an in-frame mutated allele. For Mutant 4, a wild type and three mutated *FAE1* alleles predicted to have disrupted the open reading frames were discovered. In *FAD2*, Mutant 4 had a wild type allele and a mutated allele predicted to have disrupted the open reading frame. No transgene insertions were detected at the cut sites or by *Cas9* PCR analysis (**Supplementary Figure S2**).

#### 3.2.2 Identification of *rod1* mutants

**Figure 2:**
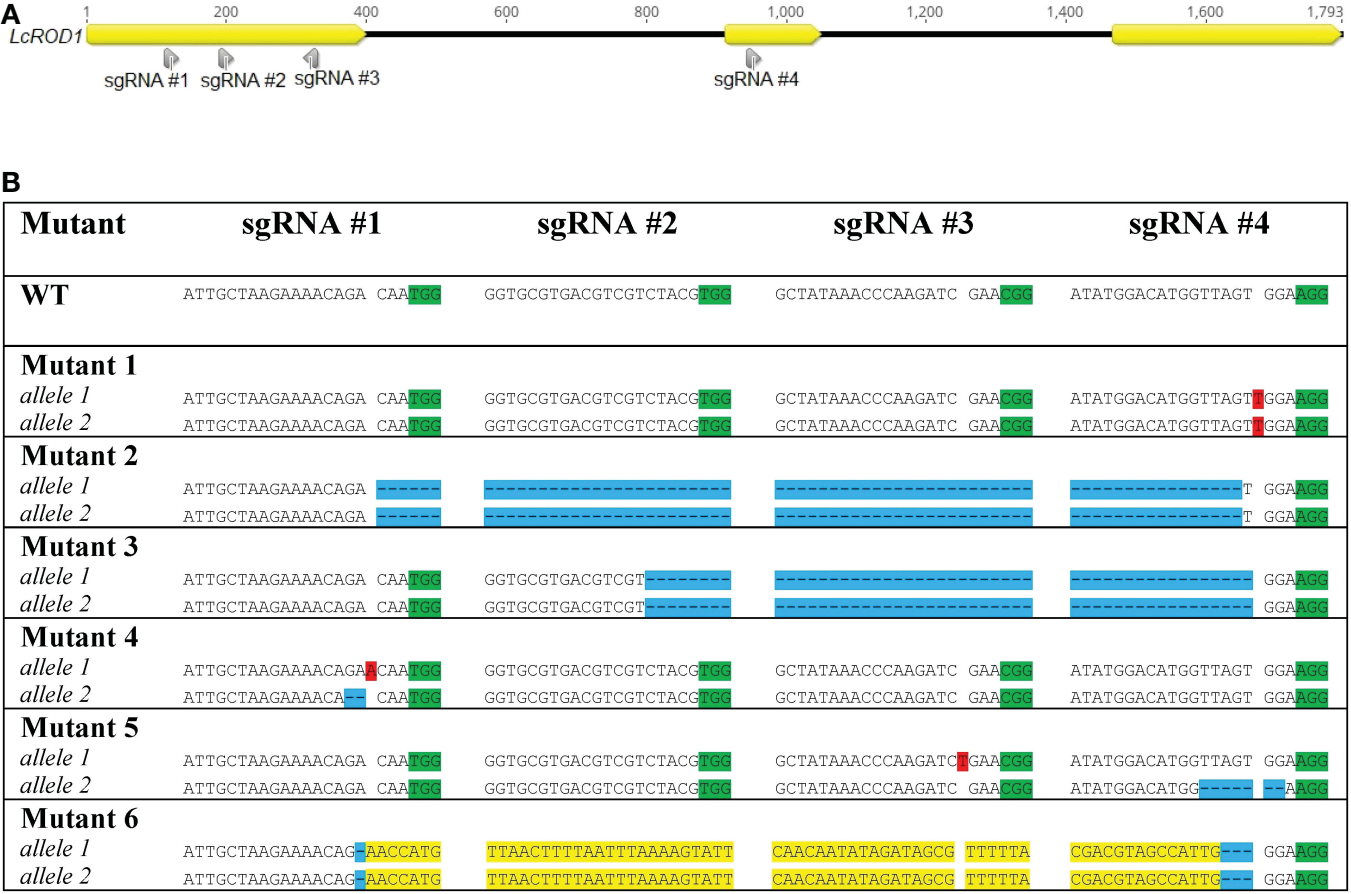
Locations of sgRNAs selected **(A)** and types of mutations detected in *ROD1* in different mutant lines **(B)**. Insertions are highlighted in red. Deletions are highlighted in blue. Inversions are highlighted in yellow. PAM sites are highlighted in green.

Ten mutated lines were identified among the 127 regenerated T_0_ regenerated shoots analyzed by HRFA, indicating 7.9% mutation efficiency. The sequencing results confirmed six distinct mutated genotypes, named Mutant 1, 2, 3, 4, 5, and 6 (**Figure 2**). All four sgRNAs were found to be able to induce mutations in *ROD1*. Both alleles in all six genotypes carried mutations predicted to disrupt the open reading frame, and the mutations were homozygous in four of the six genotypes. Mutants 4 and 5 had small heterozygous indels in both alleles, while Mutant 1 carried a homozygous +T insertion. In Mutant 2 and 3, large deletions of 830 and 756 bp, respectively, were detected. In Mutant 6, the 828 bp segment had been inverted and was flanked by small deletions. No transgene insertions were detected at any cut sites, confirmed by sequencing and *Cas9* PCR analysis (**Supplementary Figure S3**).

### 3.3 Phenotyping results by fatty acid composition analysis

#### 3.3.1 Knockout of *FAE1* resulted in significant increase in C18:1 and reduction in C22:1

**Figure 3:**
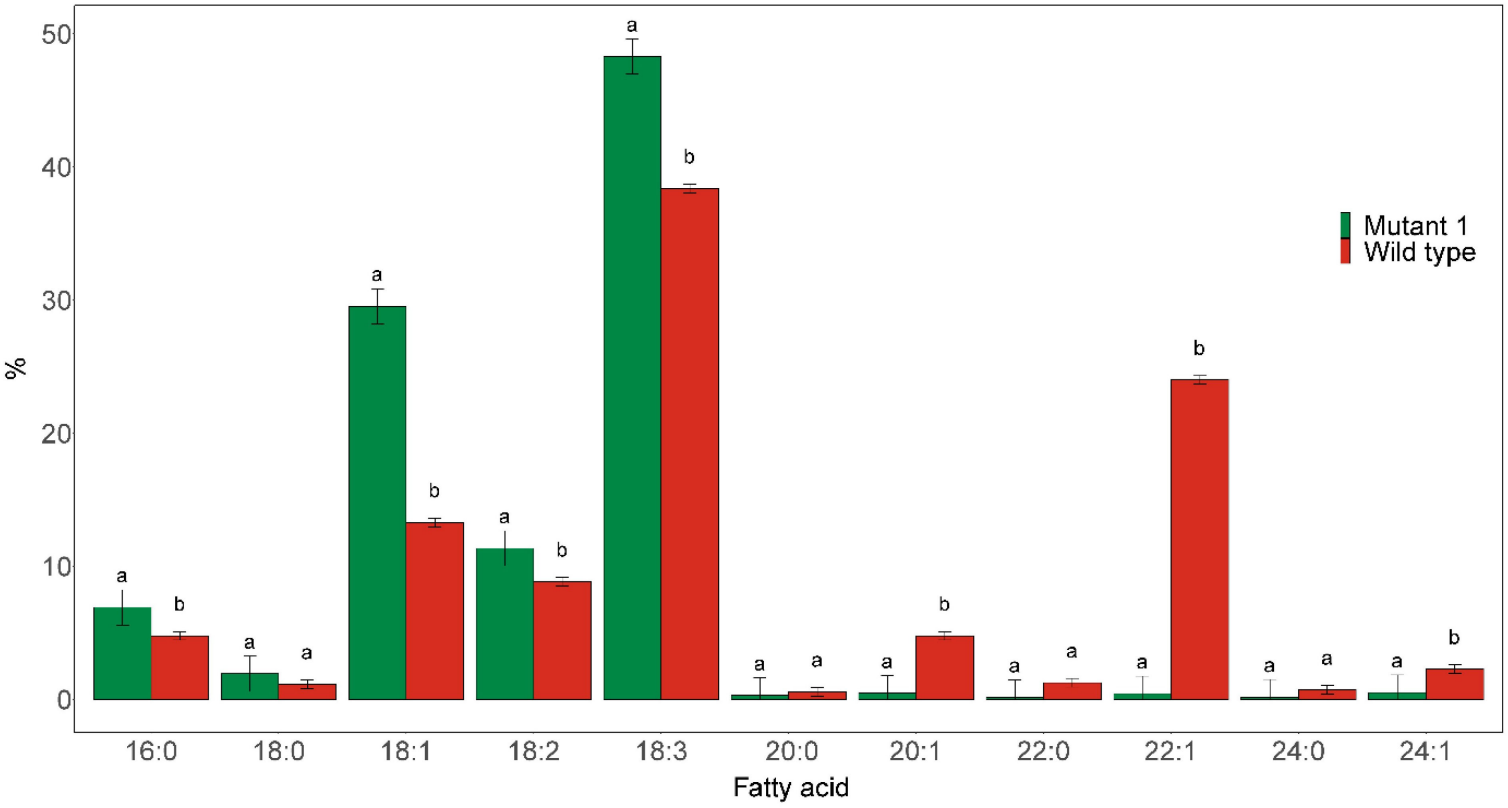
Major fatty acid compositions of seed oil derived from the seeds of homozygous *fae1* Mutant 1, as shown in **Figure 1**, and wild type. Different letters indicate statistically significant differences at *p* < 0.05 (*n* = 3).

The homozygous *fae1* Mutant 1 line was found to almost completely blocked elongation of C18:1 (**Figure 3**). Only trace amounts (≤0.5%) of C20:0, C20:1, C22:1, C24:0, and C24:1 could be detected in the seed oil of this mutant. Furthermore, the proportions of C18:1 level in the mutated line was more than doubled, from 13.3% in the wild type to 29.5% in the mutant line. The proportion of C18:3 was also significantly increased in the mutant, from 38.4% in wild type to 48.3% in the mutant.

#### 3.3.2 Mutations in *FAD2* and *FAE1* significantly increased C18:1 and reduced C18:3

**Figure 4:**
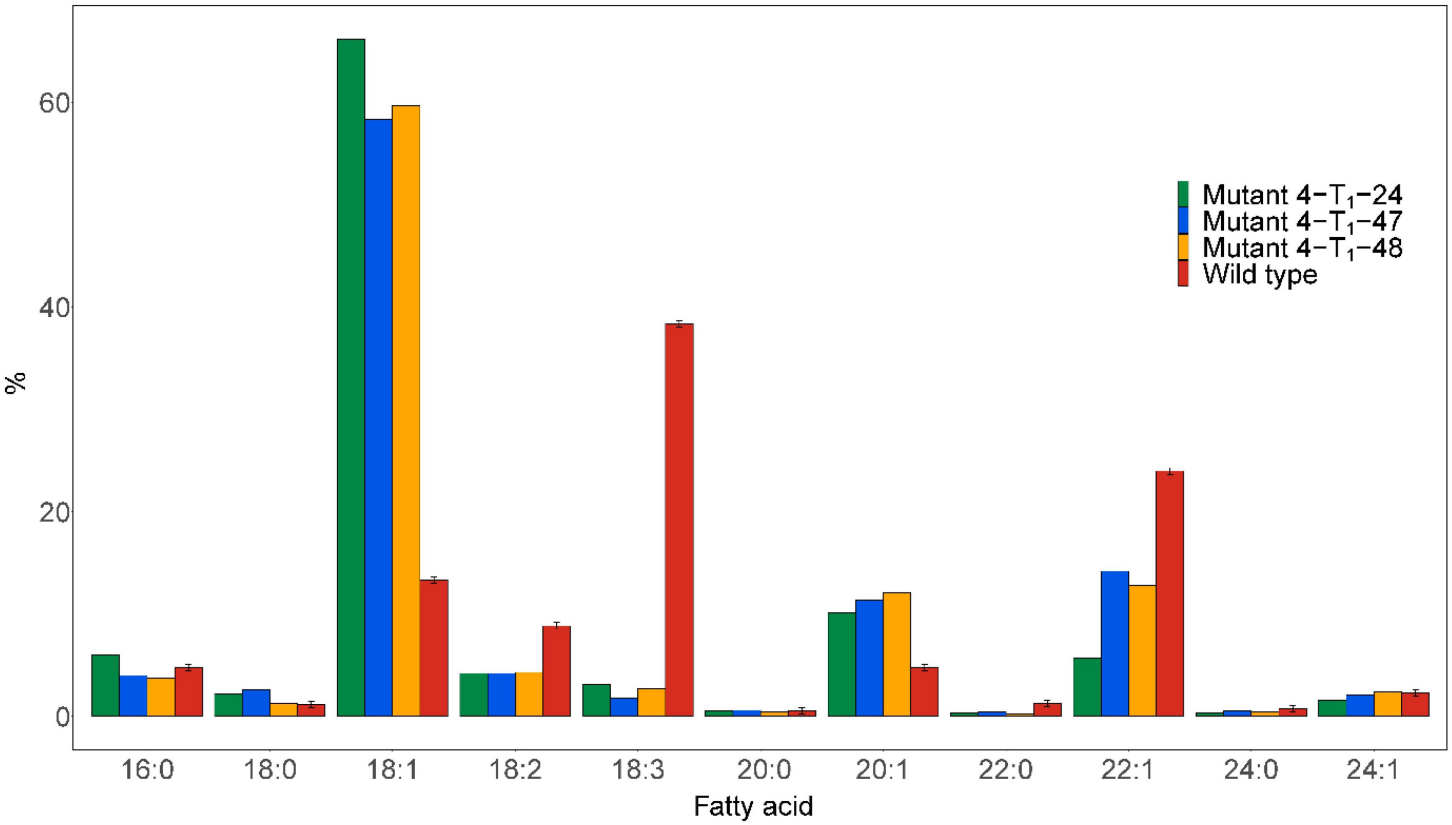
Major fatty acid compositions of seed oil from the individual seeds analyzed by the half-seed method exhibiting the most pronounced changes in fatty acid profile. Seeds were derived from selfing the heterozygous Mutant 4, as shown in **Figure 1**.

No homozygous *fad2* or double *fae1fad2* mutants were detected in T_0_. The heterozygous Mutant 4 T_0_ with *FAE1fae1* and *FAD2fad2* was thus selfed to obtain homozygous knockout lines in T_1_. Half-seed analysis was carried out on 84 T_1_ seeds derived from selfed Mutant 4. The results showed that three seeds were found to have dramatically improved fatty acid compositions, named Mutant 4-T_1_-24, Mutant 4-T_1_-47, and Mutant 4-T_1_-48 (**Figure 4**). The C18:1 content was increased from 13.3% in the wild type to 66.2%, 58.3%, and 59.7% in the mutant seeds, respectively. Furthermore, the C18:3 content was reduced from 38.4% in the wild type to 3.1%, 1.8%, and 2.7% in the mutant seeds, respectively. The C22:1 content was reduced from 24% in the wild type to 5.7%, 14.2%, and 12.8% in the mutant seed, respectively, indicating some *FAE1* activity. Moreover, the C20:1 content was increased from 4.8% in the wild type to 10.1%, 11.4%, and 12.1% in the mutant seed, respectively.

**Figure 5:**
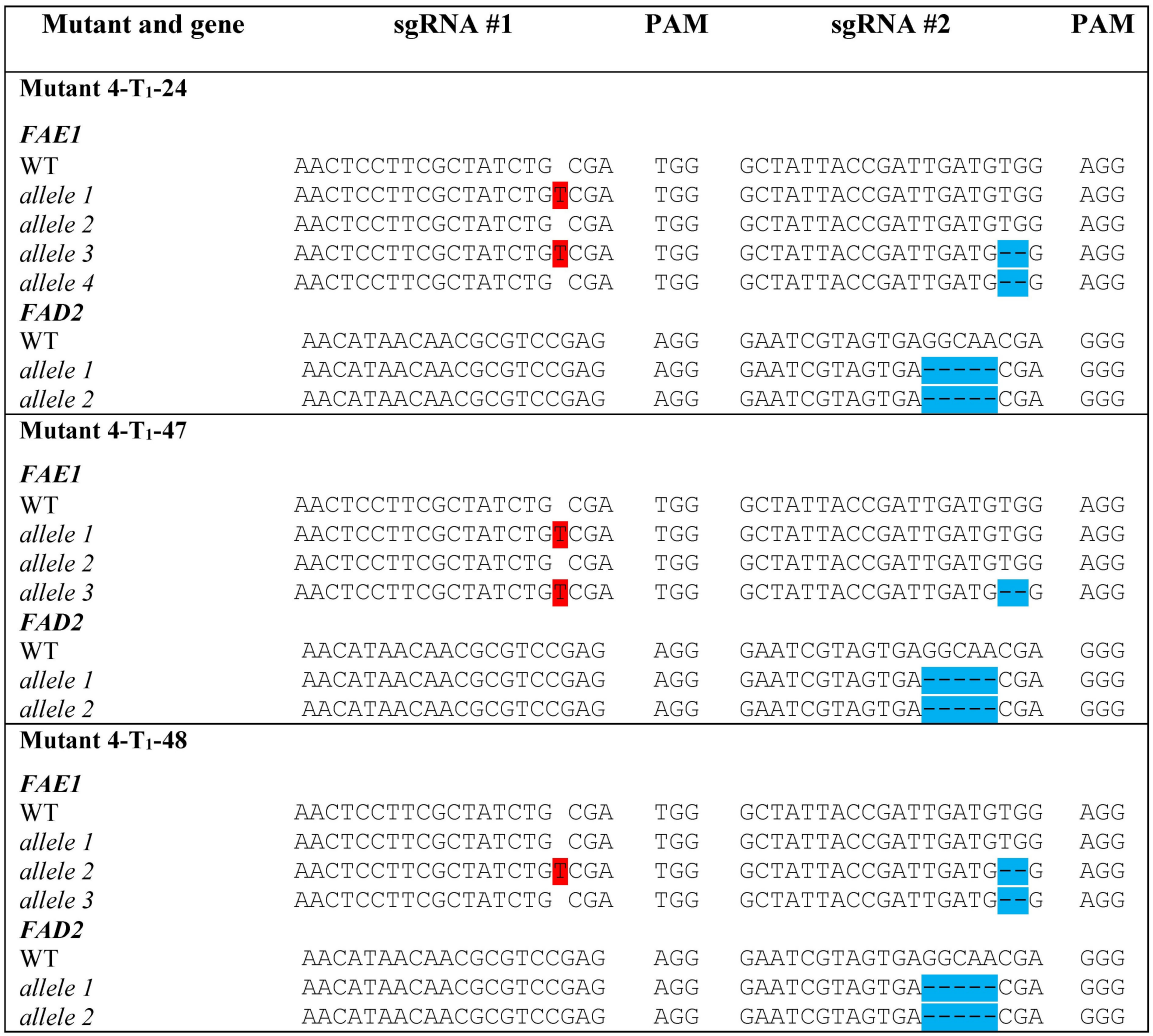
Types of mutations detected in *FAE1* and *FAD2* in selected seeds from the T_1_ generation of Mutant 4. Insertions are highlighted in red. Deletions are highlighted in blue.

Sequencing of the mutant lines revealed homozygous 5 bp deletions in *FAD2*, predicted to disrupt the open reading frames, and heterozygous mutations in *FAE1*, including a wild type *FAE1* allele (**Figure 5**).

**Figure 6:**
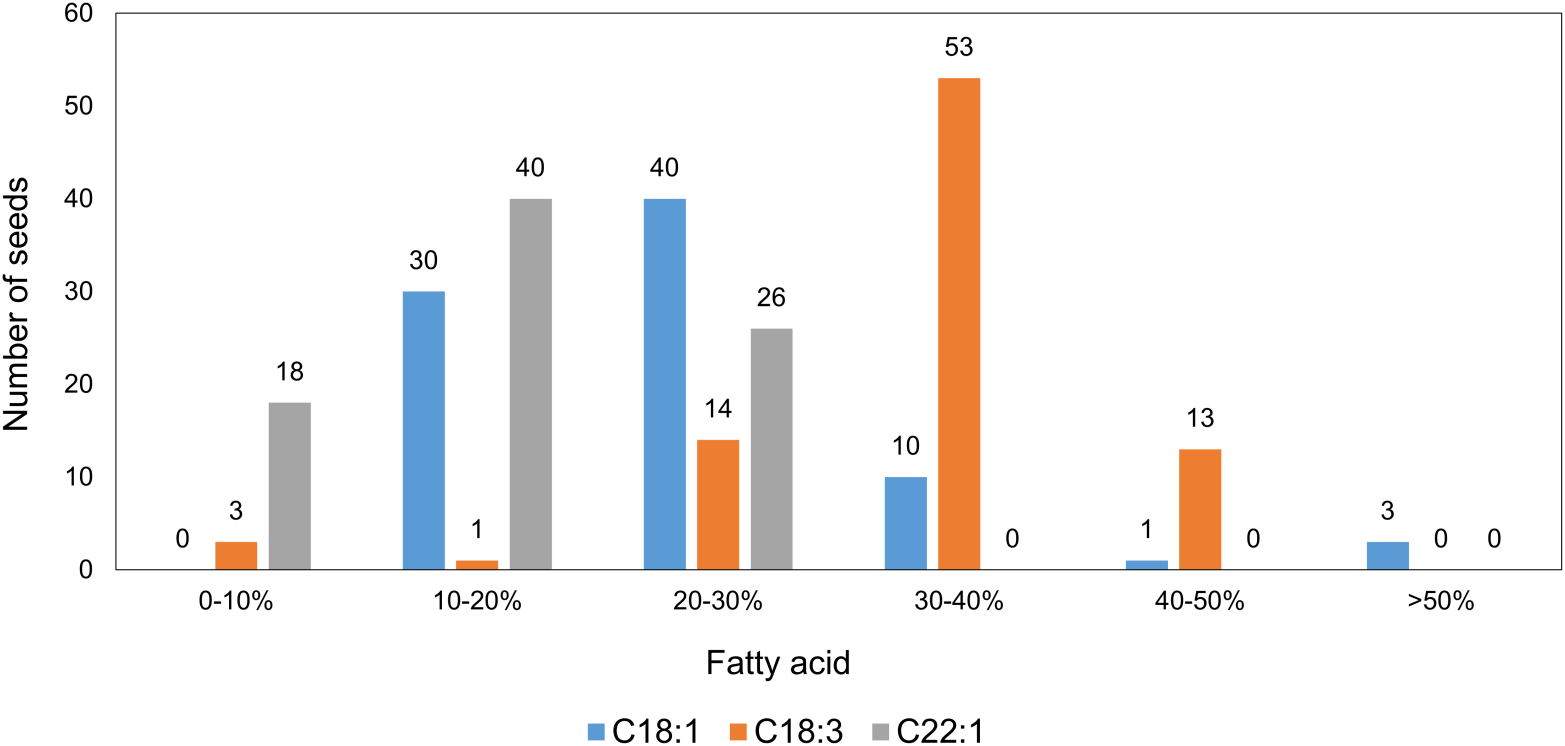
Distribution of major fatty acids in individual seeds from the T_1_ generation of Mutant 4, analyzed by the half-seed method (total seed number = 84).

The distribution of the fatty acid profiles among the 84 T_1_ seeds from Mutant 4 showed that more than half of the seeds had markedly elevated C18:1 contents, indicating that at least one *FAE1* and/or *FAD2* allele had been knocked out. Furthermore, it seemed like multiple knockout alleles had an additive effect on increasing the C18:1 content (**Figure 6**).

#### 3.3.3 Mutations in *ROD1* increased C18:1 and reduced PUFAs

**Figure 7:**
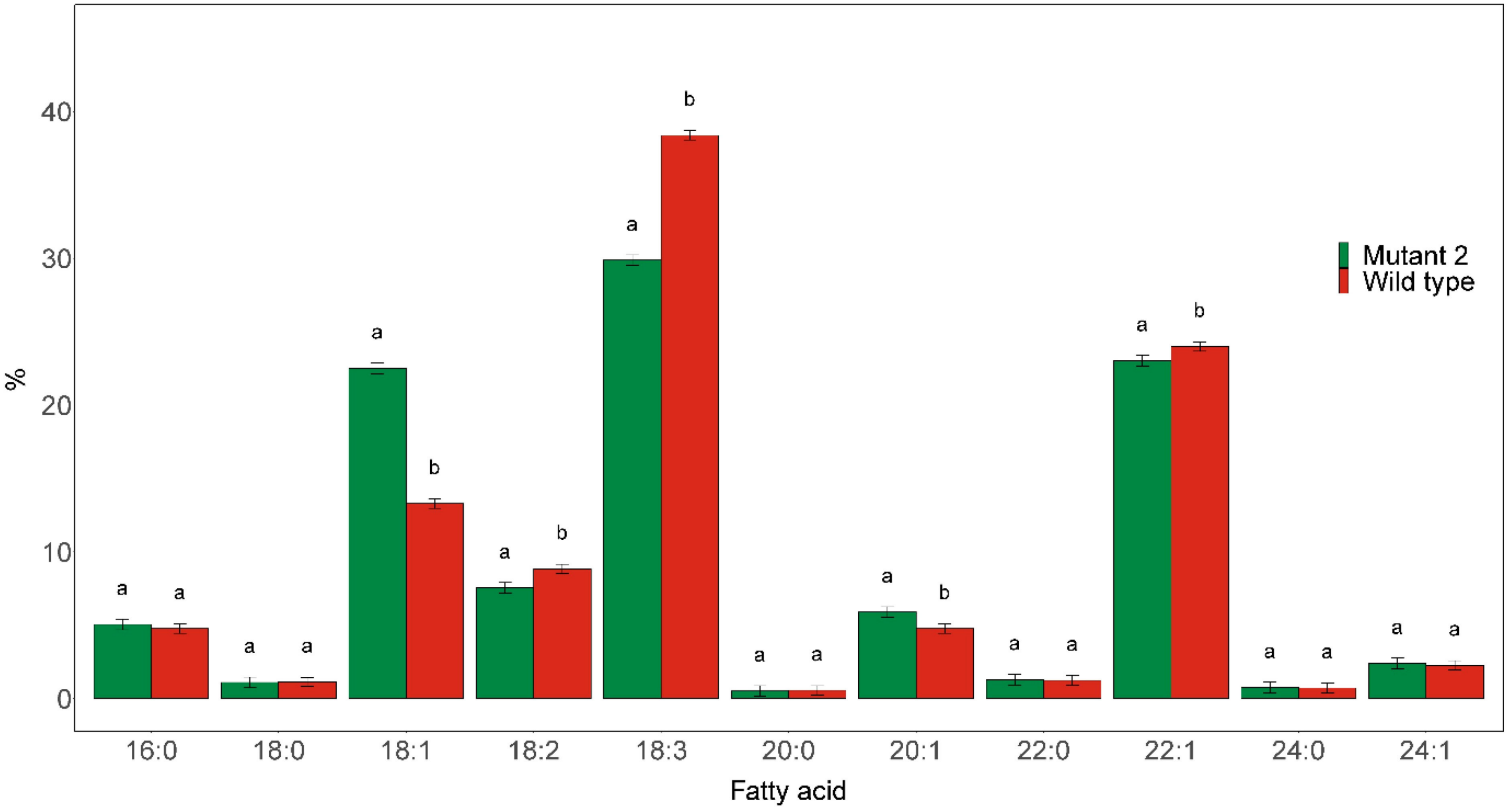
Major fatty acid compositions of seed oil derived from *rod1* Mutant 2, as shown in **Figure 2**, and wild type. Different letters indicate statistically significant differences at *p* < 0.05 (*n* = 3).

Preliminary fatty acid composition analysis of the different mutant *rod1* lines showed that they all had highly similar fatty acid profiles. We thus present only the results from Mutant 2 for simplicity. The mutant was found to have a significantly elevated C18:1 level and reduced PUFA contents (**Figure 7**). The C18:1 level was significantly increased to 22.5% compared with 13.3% in the wild type, while the C18:3 and C18:2 contents were significantly reduced from 38.4% in the wild type to 29.9% in the mutant and from 8.8% in the wild type to 7.5% in the mutant, respectively.

## 4 Discussion

Traditionally, domestication of wild plant species is highly time-consuming and laborious, especially for species such as field cress with a relatively long generation time. Modern breeding technologies have brought new perspectives in speeding up the domestication process. In this regard, the latest genome editing technique CRISPR/Cas9 holds great promise to rapidly speed up the process, as many of the key target traits can be simultaneously improved. Furthermore, many important domestication related key genes are already characterized in field cress or closely related species. The application of CRISPR/Cas9 in domestication efforts has been demonstrated in a few species, such as *Thlaspi arvense* (pennycress) (Jarvis et al., 2021), *Solanum pimpinellifolium* (wild tomato) (Li et al., 2018; Zsögön et al., 2018), *Solanum peruvianum* (wild tetraploid tomato) (Lin et al., 2022), and *Physalis pruinosa* (groundcherry) (Lemmon et al., 2018).

In order to test the feasibility of the CRISPR/Cas9 gene editing technique in modifying important traits in field cress, and to develop transgene-free mutated lines with improved oil quality - high C18:1 and low PUFAs and C22:1, we knocked out three key fatty acid biosynthesis genes using our recently developed protoplast protocol (Sandgrind et al., 2021). In field cress, it has been previously shown that knockdown of *FAE1* and *FAD2* using the RNAi approach resulted in lines that were high in C18:1 and low in PUFAs and C22:1 (Ivarson et al., 2016). However, there are certain drawbacks to the transgenic approach for commercial applications. In some countries, there are no or limited regulatory obstacles for transgene-free gene-edited plants than transgenic plants. Moreover, the vector DNA may disrupt endogenous genes if they are integrated into the plant genome, the continued expression of CRISPR vectors may increase the risk of off-target mutations, and the out-crossing of the CRISPR vectors requires additional efforts. As such, we explored the feasibility of using the protoplast-CRISPR approach for knocking out the *FAE1*, *FAD2* and *ROD1* genes in field cress in this study.

The results showed that we successfully obtained mutated lines with the desired phenotype without integration of foreign DNA in the target regions using the protoplast-CRISPR approach. As expected, full knockout of *FAE1* almost completely blocked further chain elongation from C18:1; only trace amounts (≤0.5%) of C20:1 and C22:1 were detected in the seed oil of the homozygous *fae1* mutant. Because no homozygous *fad2* or double *fae1fad2* mutant lines were obtained in T_0_, the seeds derived from the heterozygous *FAE1fae1FAD2fad2* Mutant 4 were subjected to half-seed analysis to detect potential homozygous mutant lines in T_1_ with improved oil quality. The segregation in T_1_ did not follow a normal Mendelian pattern based on the fatty acid profile results, apparently due to the chimeric nature of Mutant 4 which showed four distinct *FAE1* alleles in T_0_. More generations are needed to obtain the double homozygous mutations in *fae1fad2*. Among the 84 seeds analyzed, three contained very low amounts of C18:3, indicating complete knockout of *FAD2*, which was confirmed by the sequencing result where homozygous *fad2* alleles were found in the lines. PCR analysis with several primer pairs followed by sequencing has previously indicated a single copy of the *LcFAE1* and *LcFAD2* genes, as also shown in *A. thaliana* (Eriksson and Merker, 2011). The mutants with homozygous *fad2* alleles and heterozygous *FAE1fae1* alleles had dramatically improved oil quality (C18:1 of 58-66%, and less than 5% C18:2 and C18:3). It is expected that the C18:1 content could be further improved in homozygous *fae1fad2* lines, as the lines still contained substantial amounts of C20:1 and C22:1 that could be shifted towards C18:1, as reported in the double knockdown lines by Ivarson et al. (2016), in which the highest C18:1 level reached up to 80%.

Previous studies on *ROD1* knockdown or knockout have resulted in an increase in C18:1 and a decrease in PUFAs in the seed oil of several oilseed species. Disruption of the *LcROD1* open reading frame resulted in multiple homozygous mutant lines with an altered fatty acid profile - a modest increase in C18:1 and reduction in PUFAs, as previously shown in *A. thaliana* (Lu et al., 2009), *C. abyssinica* (Guan et al., 2015), *B. napus* (Bai et al., 2020), and *T. arvense* (Jarvis et al., 2021). However, an increase in C18:1 from 13.3% in the wild type to 22.5% in the mutant lines and a reduction in C18:3 from 38.4% in the wild type to 29.9% in the mutant lines is still very interesting for breeding purposes. This is particularly important when considering the potential adverse effects on the growth and development of *fad2* mutants in pennycress (Jarvis et al., 2021). However, for our field cress mutant lines, no clear phenotypic alterations, such as growth vigor, flowering time, or fruit setting were observed, which could be related to the mutations of the target genes.

Our results have shown that CRISPR/Cas9 could successfully induce targeted mutations at multiple target sites simultaneously in field cress, which could potentially be applied to even larger multiplex gene editing efforts in order to improve multiple traits of interest simultaneously. For all three target genes, we were able to induce mutations at all target sites, indicating that the expression of the CRISPR/Cas9 constructs along with the chosen sgRNAs worked well in this study. The mutation efficiencies obtained in this study is likely underestimated, as the HRFA method used for primary screening of mutation lines cannot detect substitutions or combination of mutations that do not result in changes in the amplified DNA fragment size. As shown in other studies, the mutation efficiency can vary a lot between species and genotypes and is greatly impacted by multiple known and unknown factors such as sgRNA, promoters, CRISPR vector delivery method and so on (Ma et al., 2015; Lin et al., 2018). Considering the relatively simple, cheap and high-throughput nature of the HRFA method in combination with a large number of shoots obtained from a single protoplast transfection, the system is highly recommended for the initial screening of mutation lines.

Insertions of vector DNAs at the target sites have been shown to be a potential issue in some species when using a vector-based CRISPR/Cas9 system (Andersson et al., 2018). In our case, no insertions of foreign DNA could be detected when analyzing the target sites by HRFA or sequencing, or by PCR analysis of the *Cas9* gene. However, it would be necessary to perform whole genome sequencing to verify that the mutated lines are truly transgene-free. To avoid this issue, our group has recently switched to the DNA-free method for delivering CRISPR/Cas9 complexes in the form of ribonucleoprotein complexes (RNPs) into plant cells. This method is supposed to be as efficient as vector-based expression systems (Chen et al., 2019). It is even more efficient according to our ongoing studies in rapeseed in terms of mutation efficiency.

In conclusion, we have demonstrated for the first time that CRISPR/Cas9 can induce targeted mutations in field cress using the protoplast approach, multiple target sites can be mutated simultaneously, and the mutated protoplasts can be regenerated into plants that set seed. Furthermore, we have demonstrated that it is possible to dramatically improve the seed oil fatty acid profile of field cress by knocking out the key genes involved in fatty acid biosynthesis. By knocking out *FAE1* it is possible to obtain a seed oil well below the maximum allowed threshold of the erucic acid level for foodstuffs in the European Union. Knockout of *FAE1*, *FAD2*, and *ROD1* increased the proportion of C18:1 in the seed oil. By stacking these knockout variants, it could be possible to create field cress lines with an exceptionally high C18:1 content, and only trace amounts of PUFAs and C22:1. In future studies, careful evaluation of the agronomic performance and seed oil content of such lines under field conditions would be of great interest and a big step forward regarding the domestication of this species, as field cress lines with improved seed oil profiles are highly desirable for food, feed, and industrial uses. This study provides a sound base for using the protoplast-CRISPR approach to modify other important traits in field cress in order to speed up the domestication process and thus make it a viable crop for temperate climates.

## Data availability statement

The datasets presented in this study can be found in online repositories. The names of the repository/repositories and accession number(s) can be found in the article/**Supplementary Material**.

## Author contributions

L-HZ led the research and, together with SS and XL, designed the experiments. EI performed cDNA synthesis of *FAE1* and *FAD2* genes. SS cloned and sequenced the genes. SS and SK generated the CRISPR/Cas9 constructs. SS performed the protoplast transfections. SS, XL, and RG performed subsequent *in vitro* tissue culture work and took care of plants in biotron. SS and EW performed the mutant screening and the fatty acid composition analysis. SS and L-HZ wrote the manuscript. All authors contributed to the article and approved the submitted version. 
