# Corrigendum: Improved fatty acid composition of field cress (*Lepidium campestre*) by CRISPR/Cas9-mediated genome editing

**DOI:** 10.3389/fpls.2023.1183817

**Published:** 2023-03-22

**Authors:** Sjur Sandgrind, Xueyuan Li, Emelie Ivarson, Eu Sheng Wang, Rui Guan, Selvaraju Kanagarajan, Li-Hua Zhu

**Affiliations:** Department of Plant Breeding, Swedish University of Agricultural Sciences, Lomma, Sweden

**Keywords:** CRISPR-editing, field cress, oil quality, protoplast, transgene-free mutants

In the published article, there was an error in the **Funding** statement. The **Funding** statement for “FORMAS -The Swedish Research Council for Sustainable Development” missed the parentheses for the full name of FORMAS and grant number. The correct **Funding** statement appears below:

“Financial support to this research by MISTRA (The Foundation for Strategic Environmental Research) and The Swedish University of Agricultural Sciences through Mistra Biotech program, TC4F (Trees and Crops for the Future), a strategic research area at SLU, supported by the Swedish Government, FORMAS (The Swedish Research Council for Sustainable Development) grant 2016-01401, and Einar and Inga Nilsson’s foundation as well as The Royal Physiographic Society of Lund is highly acknowledged.”

The authors apologize for this error and state that this does not change the scientific conclusions of the article in any way. The original article has been updated.

