# Supplementary material for: Improved fatty acid composition of field cress (Lepidium campestre) by CRISPR/Cas9-mediated genome editing
Source: Front Plant Sci. 2023 Jan 18;14:1076704. doi: 10.3389/fpls.2023.1076704 (PMC9901296; doi:10.3389/fpls.2023.1076704)
Supplement: Supplementary file 1 [file DataSheet_1.pdf]

Supplementary figures and tables

ATGTCAGCCGCCGCAACTAAAACCGTCGTCCCTCGACGTCGCAGATCTACCTCTCTTAACGGAGCTCA
CATTAACGGCGTCGAAGATACAAACACTCAAATGGAGACGAACATTGCTAAGAAAACAGACAATGGCT
ACGTCGCTAACGGAGGAGGATGGAGAAACCAAGCGTCGTTTATGACATGGACGGTGCGTGACGTCGTC
TACGTGGCGAGATATCATTGGATACCATGTTTATTCGCTGCCGGGCTTCTGTTCTTCATGGGCGTGGA
GTATACGCTCCAAATGATTCCGGCGAGATCTGAGCCGTTCGATCTTGGGTTTATAGCCACGCGCTCTT
TGCATCGCGTCTTGACATCTTCACCAGATCTTAACACTCTTTTAGCCGCTTTAAACACGGTATGTCGT
GCAAGTTAATTTAGGTTAAAAATATATTTATATTTTATGATTATCCTCAAATTCCTCTTGCCTCTTTC
ACCTAATATTTTGTTTTTTATCTGGCCTTTGTCATTTAAATCTTAATAATTTAATTGAATTAGTCAAA
ATGAAAATAAAAGTGGGGTAGAGATGGAAACAAACCACAGATTTTTTTCCTAAGCTATTTAATTCTCC
AGCTGGAATTATGCTGTCAACGCAATGAAAATATCCAAAGAGTCTTAATCTAAAATGGTCTTAATCTA
TTAATTAGTGGATAAAAATGTATTTAATGTAATCTCTTTGTTTAGTTGTAGTAGTTAATGTCCTTTCT
AGTAGTACAACAATATAGATAGCGTTTTTAACTTTAGAACACCAAATAAAAAAGACACGTGGTGATGA
GTTGGAGATCGACATGATCACATGCATACAAAGAGATGGGTTAAATTCAAGTCTTGTAATACTTTTAA
ATTAAAAGTTAATTGAATGTGGGGACAGGTGTTCGTAGGGATGCAAACAACGTATATAATATGGACAT
GGTTAGTGGAAGGACGAGCACGAGCCACCATCTCTGCTTTATTCATGTTCACTTGTCGTGGTATTCTC
GGTTACTCTACTCAGCTCCCACTTCCTCAGGTTCTAATCCACTTATCTAATTACTTCTATTGAATAGT
GTGAAAGGTCTAATATATAGTATTTAACGCCTTTAAGTTGCGGGATTTAGGTTAATTATCATCATCTT
AATGATTGTATTTCAATTTTCAGACATGACCGTTTTGTTTTCTTTTGTTACTGTATTTTTTTCAATCT
TCCAAAAAAATGTTCCACGATCTGATTGAGTGGACATAAATATCAGATTCTCACTCATTTACGTTATA
CGGCTTTAGTGACTCATCTATTTATTATCTTTCTCGTGATATCTAGCATAGGTACATGTTTTTACGTG
TCTTCTTGTTATACGAATCAAGAAAAAAACAAAGATTTTAGGTTGATTAATTATAGCTTTAGTAATCA
AGATTAGCTGATCTTGGAATTACATGATTCTTTTGCAGGAATTTTTAGGATCAGGAGTGGATTTTCCA
GTGGGAAACGTCTCATTCTTCCTCTTCTTCTCTGGCCATGTCGCTGGCTCGATGATTGCGTCCTTGGA
CATGAAGAGAATGCAAAGGTTTAGACTTGCGATGGTTTTTGACATCCTCAATGTATTACAATCGATCA
GGCTGCTTGGTACTAGAGGACACTACACAATCGATCTTGCAGTTGGAGTTGGCGCTGGAATAATCTTT
GACTCATTGGCCGGGAAGTACGAAGAGATGAGCAAGAGTCGACACTTAAGCCCTAGTTTTAATTTGAT
TTCAAAAGATTCTCTAGTCAATTAA

Supplementary Figure S1 *LcROD1* gene sequence. Exons are highlighted in green.

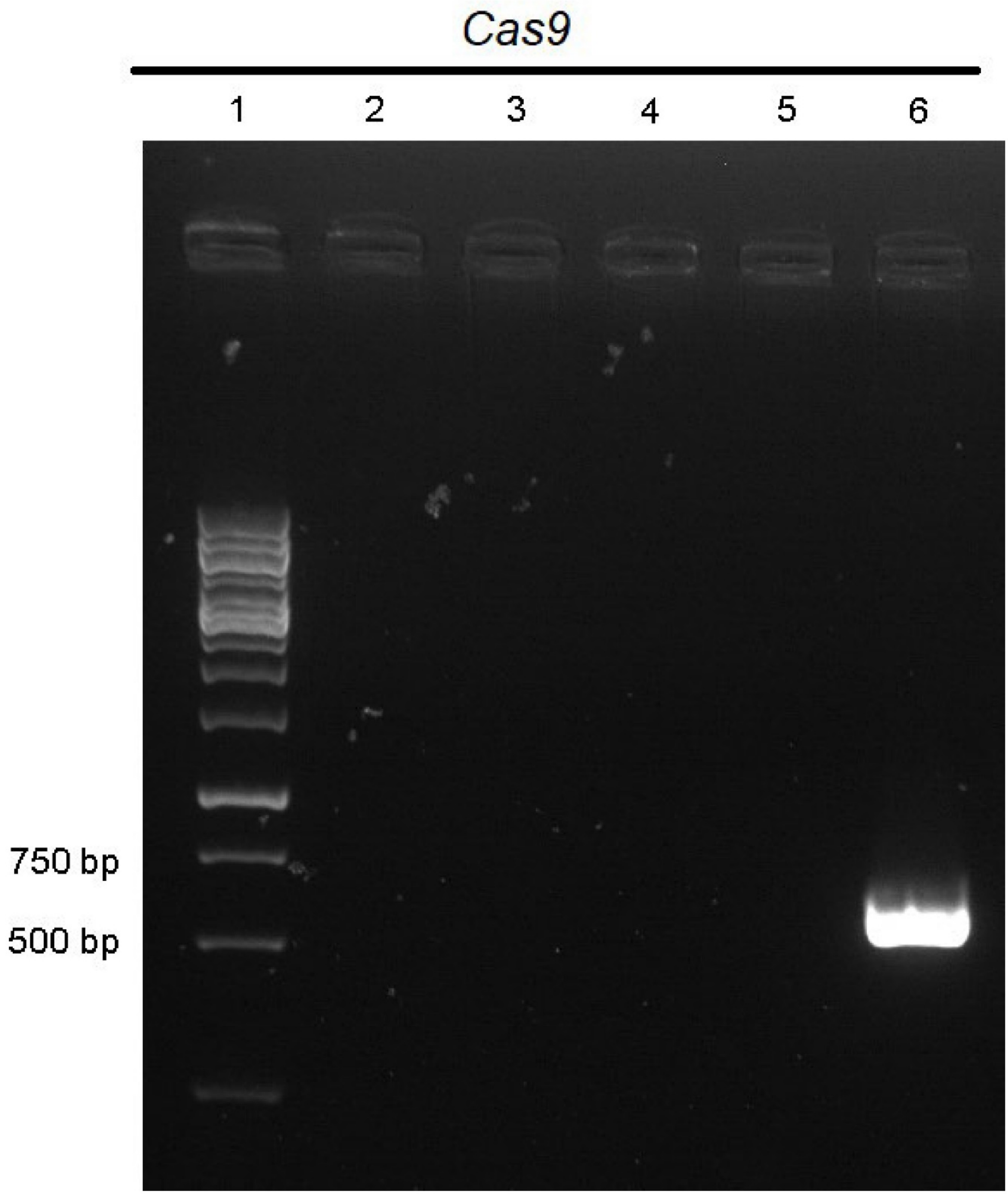

Supplementary Figure S2 PCR analysis on *Cas9* in *fae1* and *fad2* mutants, as shown in Figure 1. Lane 1, 1 kb ladder; lane 2, Mutant 1; lane 3, Mutant 2; lane 4, Mutant 3; lane 5, Mutant 4; lane 6 positive control (vector pYLCRISPR/Cas9P_{ubi}-N_FAE1/FAD2). The PCR product was 567 bp of the *Cas9* gene.

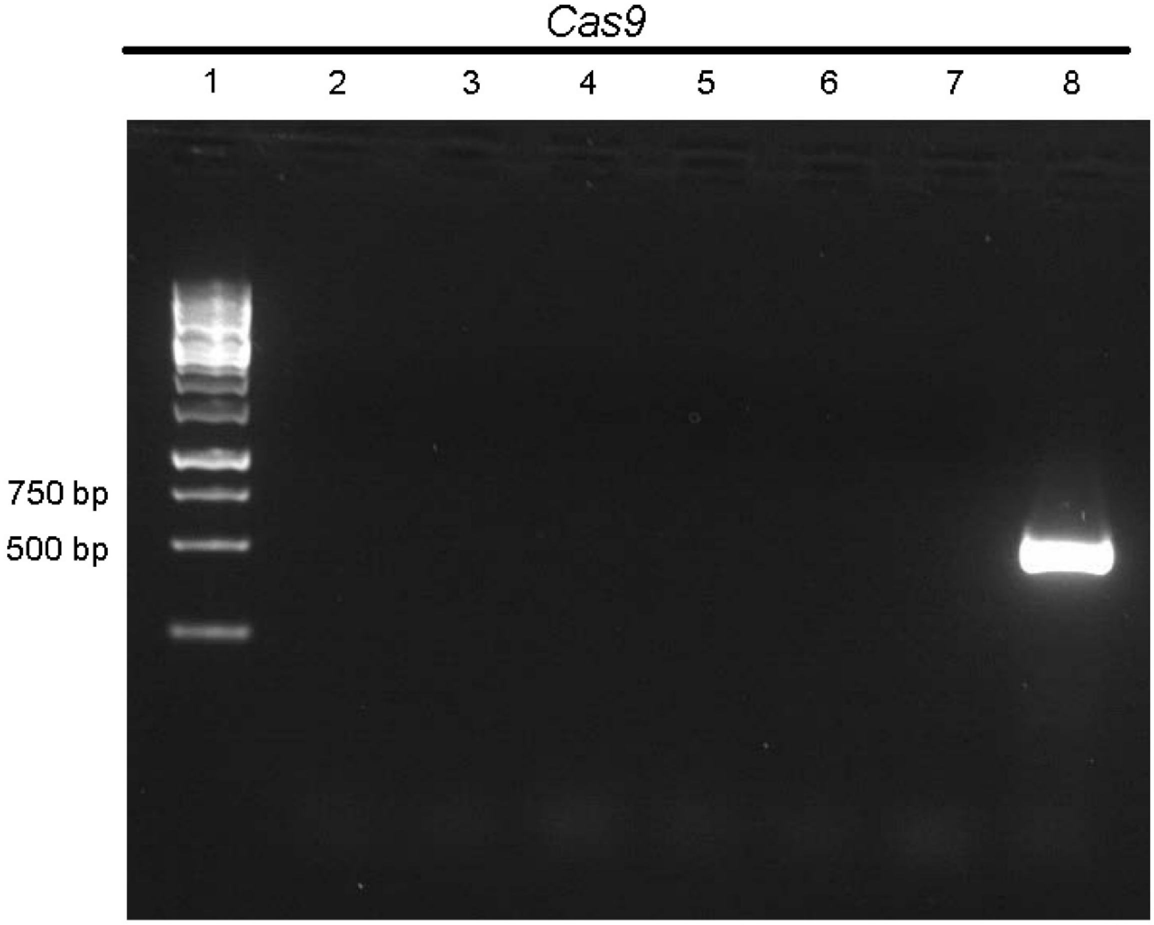

Supplementary Figure S3 PCR analysis to detect presence of *Cas9* in *ROD1* mutated lines, as shown in Figure 2. Lane 1, 1 kb ladder; lane 2, Mutant 1; lane 3, Mutant 2; lane 4, Mutant 3; lane 5, Mutant 4; lane 6, Mutant 5; lane 7, Mutant 6; lane 8 positive control (pYLCRISPR/Cas9P_{ubi}-N_ROD1). The PCR product was 567 bp of the *Cas9* gene.

Mutant 1

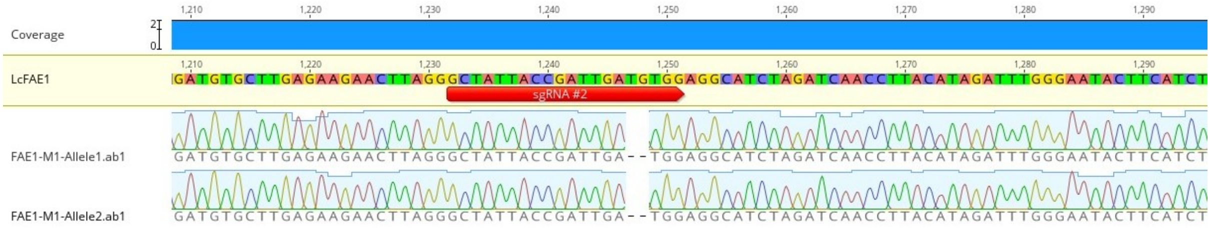

Mutant 2

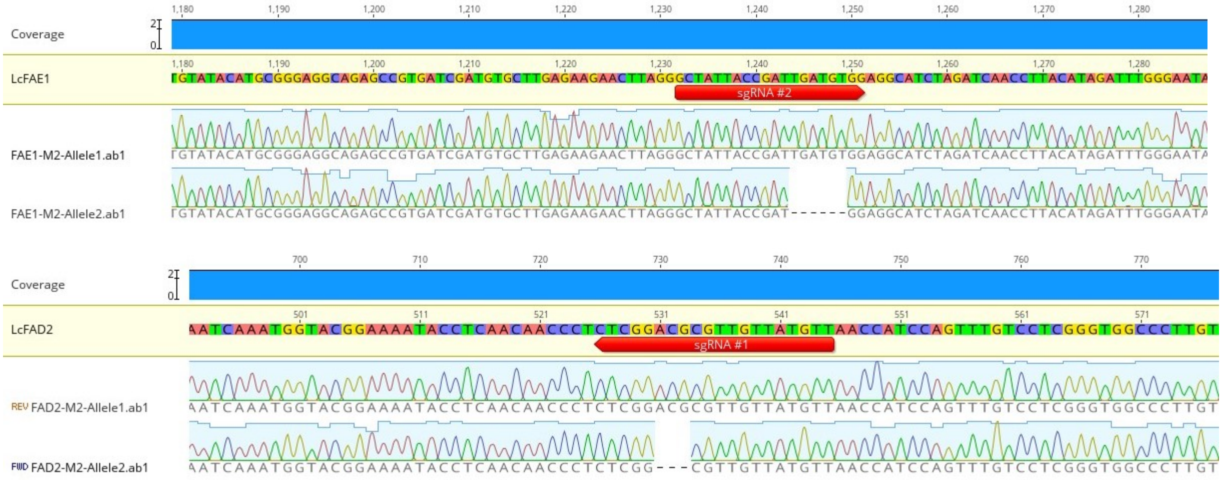

Mutant 3

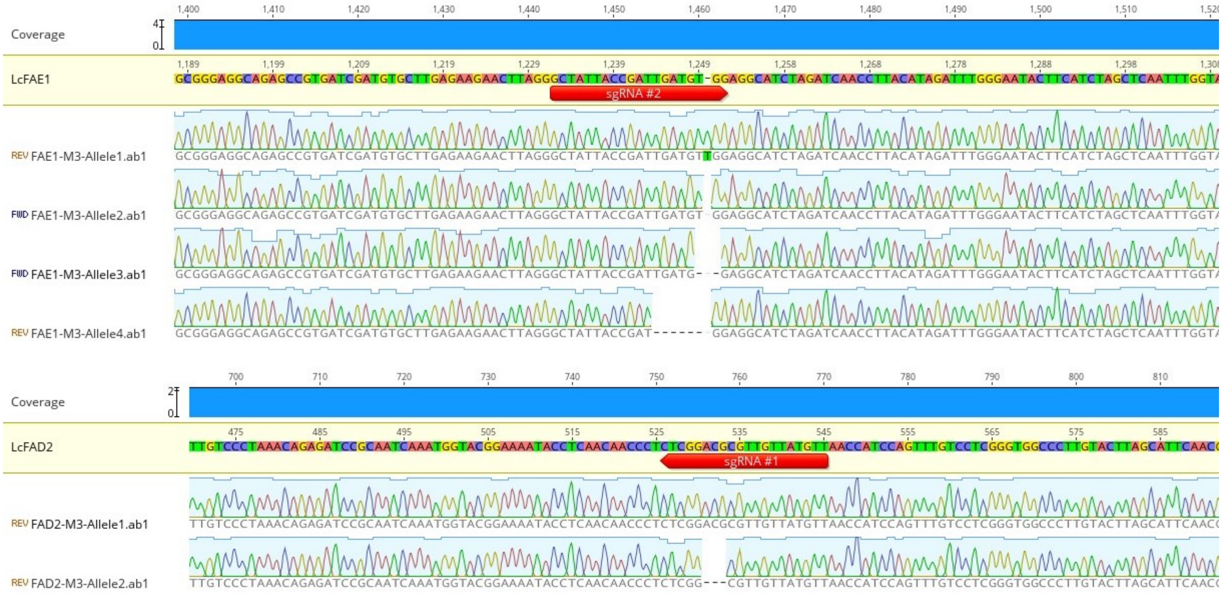

Mutant 4

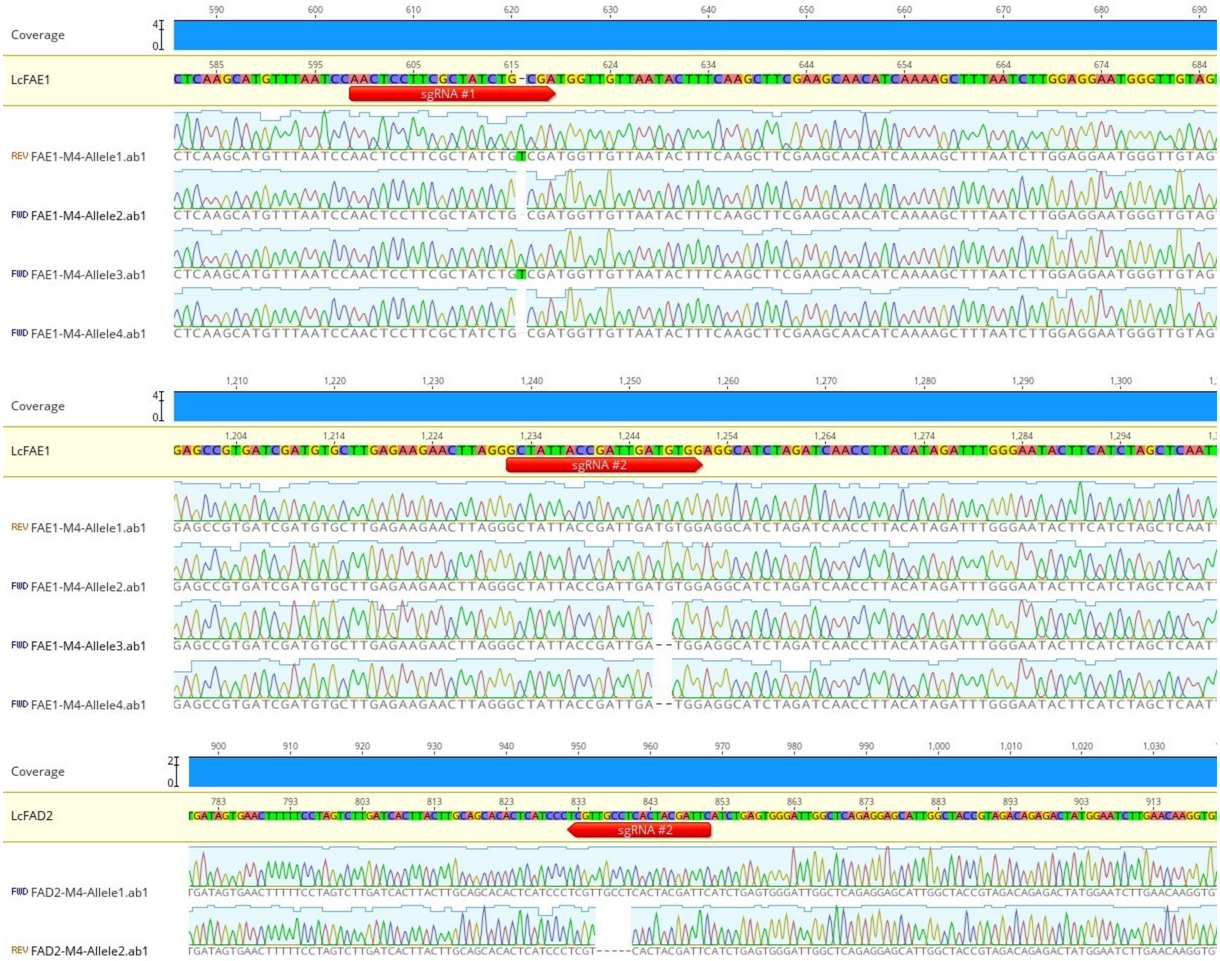

Supplementary Figure S4 Chromatograms from Sanger sequencing of the *FAE1* and *FAD2* mutant lines aligned with the wild type reference sequence.

Supplementary Table S1 List of primers used for cloning and sequencing of *LcROD1*

Primer name	Sequence (5'-3')
LcROD1-FP1	TACAAATCCAGCATAACAAGT
LcROD1-FP2	AGATCTGTTTACACGCCGGT
LcROD1-FP3	TGTCGCTCTCAAATCTCATACCA
LcROD1-FP4	CTGGAATTATGCTGTCAACGCA
LcROD1-FP5	ACGGCTTTAGTGACTCATCT
LcROD1-FP6	ACTGAATTGTGGAATAGTGGAGA
LcROD1-FP7	TTGTCGCTCTCTCATCTCGT
LcROD1-FP8	TGAATCGACAATACCCGTTCCA
LcROD1-RP1	AATCAAATTACTTGGGAGATTTGT

Supplementary Table S2 CRISPR/Cas9 target sequences (sgRNAs) and sgRNA promoters

Name	Vector	Sequence (5' – 3')	Target	Promoter
FAE1 sgRNA #1	pYLCRISPR/Cas9P_{ubi}-N_FAE1/FAD2	AACTCCTTCGCTATCTGCGA	*FAE1*	AtU3d/LacZ
FAE1 sgRNA #2	pYLCRISPR/Cas9P_{ubi}-N_FAE1/FAD2	GCTATTACCGATTGATGTGG	*FAE1*	AtU6-29
FAD2 sgRNA #1	pYLCRISPR/Cas9P_{ubi}-N_FAE1/FAD2	AACATAACAACGCGTCCGAG	*FAD2*	AtU3b
FAD2 sgRNA #2	pYLCRISPR/Cas9P_{ubi}-N_FAE1/FAD2	GAATCGTAGTGAGGCAACGA	*FAD2*	AtU6-1
ROD1 sgRNA #1	pYLCRISPR/Cas9P_{ubi}-N_ROD1	ATTGCTAAGAAAACAGACAA	*ROD1*	AtU3d/LacZ
ROD1 sgRNA #2	pYLCRISPR/Cas9P_{ubi}-N_ROD1	GGTGCGTGACGTCGTCTACG	*ROD1*	AtU6-1
ROD1 sgRNA #3	pYLCRISPR/Cas9P_{ubi}-N_ROD1	GCTATAAACCCAAGATCGAA	*ROD1*	AtU6-29
ROD1 sgRNA #4	pYLCRISPR/Cas9P_{ubi}-N_ROD1	ATATGGACATGGTTAGTGGA	*ROD1*	AtU3b

Supplementary Table S3 List of primers used for expression cassette construction

Primer	Plasmid	Sequence (5'-3')	Description
FAE1-1 FP	pYLsgRNA-AtU3d/LacZ	GTCAACTCCTTCGCTATCTGCGA	*FAE1* sgRNA1
FAE1-1 RP	pYLsgRNA-AtU3d/LacZ	AAACTCGCAGATAGCGAAGGAGTT	*FAE1* sgRNA1
FAD2-1 FP	pYLsgRNA-AtU3b	GTCAACATAACAACGCGTCCGAG	*FAD2* sgRNA1
FAD2-1 RP	pYLsgRNA-AtU3b	AAACCTCGGACGCGTTGTTATGTT	*FAD2* sgRNA1
FAD2-2 FP	pYLsgRNA-AtU6-1	ATTGAATCGTAGTGAGGCAACGA	*FAD2* sgRNA2
FAD2-2 RP	pYLsgRNA-AtU6-1	AAACTCGTTGCCTCACTACGATTC	*FAD2* sgRNA2
FAE1-2 FP	pYLsgRNA-AtU6-29	GCTATTACCGATTGATGTGG	*FAE1* sgRNA2

FAE1-2 RP	pYLsgRNA-AtU6-29	AAACCCACATCAATCGGTAATAGC	*FAE1* sgRNA2
ROD1-1 FP	pYLsgRNA-AtU3d/LacZ	GTCATTGCTAAGAAAACAGACAA	*ROD1* sgRNA1
ROD1-1 RP	pYLsgRNA-AtU3d/LacZ	AAACTTGTCTGTTTTCTTAGCAA	*ROD1* sgRNA1
ROD1-4 FP	pYLsgRNA-AtU3b	GTCATATGGACATGGTTAGTGGA	*ROD1* sgRNA4
ROD1-4 RP	pYLsgRNA-AtU3b	AAACTCCACTAACCATGTCCATA	*ROD1* sgRNA4
ROD1-2 FP	pYLsgRNA-AtU6-1	ATTGGTGCGTGACGTCGTCTACG	*ROD1* sgRNA2
ROD1-2 RP	pYLsgRNA-AtU6-1	AAACCGTAGACGACGTCACGCAC	*ROD1* sgRNA2
ROD1-3 FP	pYLsgRNA-AtU6-29	ATTGCTATAAACCCAAGATCGAA	*ROD1* sgRNA3
ROD1-3 RP	pYLsgRNA-AtU6-29	AAACTTCGATCTTGGGTTTATAG	*ROD1* sgRNA3

Supplementary Table S4 List of primers used for HRFA

**Primer Name**	**Gene**	**Sequence (5'-3')**
FAE1 F FAM	*FAE1*	CTTGTGGTGAACTCAAGCATGT
FAE1 R	*FAE1*	TAGAGCCACCCAAACTGCAC
FAD2 F HEX	*FAD2*	ACCATTCCAACACGGGATCC
FAD2 R	*FAD2*	GGGGCATTGTCGAGAACAGA
ROD1 F HEX	*ROD1*	CTCGACGTCGCAGATCTACC
ROD1 R	*ROD1*	TGAGGAAGTGGGAGCTGAGT

Supplementary Table S5 List of primers used for *Cas9* transgene detection.

**Primer Name**	**Gene**	**Sequence (5'-3')**
CAS9 1F	*Cas9*	CTGCTTCCATGATCAAGCGC
CAS9 1R	*Cas9*	CCTTCTCGTTGGGGAGGTTC
